# Fecal microbiota is associated with extraintestinal manifestations in inflammatory bowel disease

**DOI:** 10.1080/07853890.2024.2338244

**Published:** 2024-04-22

**Authors:** Sandra Hertz, Jacqueline Moltzau Anderson, Hans Linde Nielsen, Claire Schachtschneider, Kathryn E. McCauley, Mustafa Özçam, Lone Larsen, Susan V. Lynch, Henrik Nielsen

**Affiliations:** a Department of Infectious Diseases, Aalborg University Hospital, Aalborg, Denmark; b Department of Clinical Medicine, Aalborg University, Aalborg, Denmark; c Department of Medicine, Division of Gastroenterology, University of California San Francisco, San Francisco, CA, USA; d Department of Clinical Microbiology, Aalborg University Hospital, Aalborg, Denmark; e Department of Gastroenterology, Aalborg University Hospital, Aalborg, Denmark; f Department of Clinical Medicine, Center for Molecular Prediction of Inflammatory Bowel Disease, PREDICT, Aalborg University, Aalborg, Denmark

**Keywords:** Gut microbiota, inflammatory bowel disease, Crohn’s disease, ulcerative colitis, extraintestinal manifestations, 16S rRNA sequencing

## Abstract

**Introduction:**

A large proportion of patients with inflammatory bowel disease (IBD) experience IBD-related inflammatory conditions outside of the gastrointestinal tract, termed extraintestinal manifestations (EIMs) which further decreases quality of life and, in extreme cases, can be life threatening. The pathogenesis of EIMs remains unknown, and although gut microbiota alterations are a well-known characteristic of patients with IBD, its relationship with EIMs remains sparsely investigated. This study aimed to compare the gut microbiota of patients with IBD with and without EIMs.

**Methods:**

A total of 131 Danish patients with IBD were included in the study, of whom 86 had a history of EIMs (IBD-EIM) and 45 did not (IBD-C). Stool samples underwent 16S rRNA sequencing. Amplicon sequence variants (ASVs) were mapped to the Silva database. Diversity indices and distance matrices were compared between IBD-EIM and IBD-C. Differentially abundant ASVs were identified using a custom multiple model statistical analysis approach, and modules of co-associated bacteria were identified using sparse correlations for compositional data (SparCC) and related to patient EIM status.

**Results:**

Patients with IBD and EIMs exhibited increased disease activity, body mass index, increased fecal calprotectin levels and circulating monocytes and neutrophils. Microbiologically, IBD-EIM exhibited lower fecal microbial diversity than IBD-C (Mann–Whitney’s test, *p* = .01) and distinct fecal microbiota composition (permutational multivariate analysis of variance; weighted UniFrac, *R*^2^ = 0.018, *p* = .01). A total of 26 ASVs exhibited differential relative abundances between IBD-EIM and IBD-C, including decreased *Agathobacter* and *Blautia* and increased *Eggerthella lenta* in the IBD-EIM group. SparCC analysis identified 27 bacterial co-association modules, three of which were negatively related to EIM (logistic regression, *p* < .05) and included important health-associated bacteria, such as *Agathobacter* and *Faecalibacterium*.

**Conclusions:**

The fecal microbiota in IBD patients with EIMs is distinct from that in IBD patients without EIM and could be important for EIM pathogenesis.

## Introduction

Inflammatory bowel disease (IBD) is a group of gastrointestinal inflammatory disorders, the two major subtypes being ulcerative colitis (UC) and Crohn’s disease (CD). In Denmark, the prevalence of IBD has doubled over the past 20 years [1], with incidence rates in North Denmark in 2020 of 27.5/100,000 persons (CD: 11.5/100,000, UC: 15.9/100,000) [2]. IBD is a complex and multifactorial disease exhibiting high heterogeneity in clinical presentation, disease course and treatment response. Some individuals experience mild symptoms that can be effectively managed with minimal intervention, while others have more severe disease that is refractory to treatment [3–5]. Up to 50% of IBD patients experience extraintestinal manifestations (EIMs), which encompass a range of inflammatory conditions outside the gastrointestinal tract [6]. These include, but are not limited to, musculoskeletal (peripheral and axial arthropathies), ocular (episcleritis, uveitis), skin (erythema nodosum, psoriasis, pyoderma gangrenosum) and hepatobiliary (primary sclerosing cholangitis (PSC), autoimmune hepatitis) manifestations [7,8]. The implications of EIMs can vary from a notable decrease in patients’ quality of life to life-threatening complications, such as end-stage liver disease [6,7]. Moreover, EIM diagnosis can be challenging because symptoms can be non-specific and overlap with those of other diseases [7, 9], further complicating disease management [10].

Significant advancements have been made in understanding the pathogenesis of IBD, wherein the gut microbiota, genetic predisposition and environmental factors form a complex interplay to promote disease [11]. However, the pathogenesis of EIMs remains unclear and mechanisms driving EIM development in subsets of patients remain unknown. The challenges surrounding EIMs, along with the heightened risks they pose, such as vision loss, immobility, cutaneous scarring, cancers and liver failure, amongst others, emphasize the need to gain a greater understanding of the underlying contributors to EIMs. Gut microbiome perturbation is a well-established characteristic of IBD, characterized by lower diversity (compared with healthy subjects) and a depletion of short-chain fatty acid-producing obligate anaerobes, such as *Roseburia spp.* and *Faecalibacterium prausnitzii* and an increase in *Escherichia coli* and *Ruminococcus gnavus* [12,13]. Mar et al. found that UC patients could be stratified into four distinct groups based on the composition of their gut microbiota [14]. Notably, these groups exhibited significant differences in both disease severity and the presence of EIMs [14], providing initial evidence of a relationship between the gut microbiome composition and EIMs. A case report of a patient with CD who developed EIMs after receiving a fecal microbial transplant for *Clostridioides difficile* infection further implicated gut microbiota in EIM development [15]. More recently, the effects of the gut microbiome have been shown to extend beyond the gastrointestinal tract via microbial-derived metabolites that influence signalling at remote sites, such as the brain [16] and lungs [17,18], offering a mechanism by which the gut microbiome may elicit extra-intestinal effects. Moreover, a study investigating fecal microbiota similarities between CD (*n* = 79), spondyloarthritis (SpA) (*n* = 113) and uveitis (*n* = 112) found a shared immune-mediated disease signal characterized by a lower abundance of *Lachnospiraceae*, especially *Blautia*, compared with controls (*n* = 63) [19]. Additionally, IBD patients with PSC (*n* = 85) have been shown to exhibit a significantly distinct gut microbiota compared to IBD patients without PSC (*n* = 263) [20].

These findings highlight the potential involvement of gut microbiota in the pathogenesis of EIMs in IBD. Here, we report our initial findings, in which we sought to examine the clinical, immunological and gut microbiota relationships with EIMs in a large cohort of IBD patients.

## Methods

### Study population

A total of 156 adult patients with CD (ICD-10: K50) or UC (ICD-10: K51) from Aalborg University Hospital, Aalborg, Denmark, were included from 2020 to 2022. Based on their medical history, patients were divided into groups consisting of participants with EIMs (case group: IBD-EIM (incl. CD-EIM or UC-EIM)) or without EIMs (control group: IBD-C (incl. CD-C or UC-C)). Exclusion criteria for this study included treatment with systemic antimicrobial therapy within 30 days of inclusion, pregnancy or breastfeeding, terminal illness or dementia. EIM groups were defined as current or previous (1) diagnosis of SpA (ICD-10: M45-M46.8) or arthropathy with IBD (ICD-10: M07.1-6) and/or (2) diagnosis of secondary uveitis, non-infectious (ICD-10: H20.041-043) and/or (3) diagnosis of PSC (ICD-10: K83.01). History of other EIMs, such as skin manifestations (erythema nodosum, pyoderma gangrenosum and sweets syndrome), as well as IBD-related complications was also noted. The control group consisted of IBD patients without a history of EIMs and with IBD diagnosis for more than five years. Eligible IBD participants were identified by ICD-10 diagnosis codes and screened through answers from routine questionnaires from the outpatient IBD database ‘Gastrobio’ [21] and invited to participate by letter or during a clinic visit. The study was approved by The North Denmark Region Committee on Health Research Ethics (N-20190021) and all participants provided written consent before participation.

### Clinical data

Each patient completed a questionnaire regarding their disease history and current symptoms. Clinical data regarding diagnosis time, symptoms, treatment and previous paraclinical findings (colonoscopy, blood analysis and pathology) were collected from medical records, including the Montreal classification [22]. Self-reported disease activity scores were noted from each participant upon inclusion (Harvey–Bradshaw Index (HBI) for CD and Simple Colitis Clinical Activity Index (SCCAI) for UC). Study data were collected and managed using REDCap electronic data capture tools hosted at Aalborg University Hospital [23,24]. Outcome variables included EIM (yes/no) and the total number of EIMs ever experienced (0, 1, ≥2).

### Sample collection and measurements

Each participant provided a stool sample. Immediately after collection, the samples were cooled to 4 °C by storage in a cooling bag with cooling packs, and subsequently frozen at −80 °C within a maximum of 24 h between sampling and freezing. Fecal calprotectin (f-calprotectin) levels were measured in all study participants. Blood samples were collected within 24 h of stool sample collection. Blood analysis included C-reactive protein, leukocyte concentration including differential counts (neutrophils, lymphocytes, monocytes, eosinophils and basophils), haemoglobin, platelets, albumin, alkaline phosphatase, alanine transaminase and bilirubin.

### DNA extraction of stool samples

DNA was extracted from stool samples using a modified DNeasy 96 PowerSoil Pro QIAcube HT Kit (QIAGEN, Copenhagen, Denmark). Stool samples were placed on dry ice where a 0.5 g subsample of frozen stool was transferred into a sterile 2 mL cryotube under aseptic conditions in a biosafety cabinet using 4 mm sterile punch biopsies with plunger (Scandidact, Odder, Denmark) and were subsequently stored at −80 °C until DNA extraction. DNA was extracted using the high-through put extraction method by Jensen et al. [25]. In short, samples were thawed on ice, and 100 µL of stool (∼125 mg) from each sample was transferred to a 1.2 mL matrix tube prefilled with Lysing Matrix E (MP Biomedicals, Santa Ana, CA). Then, 500 µL of CD1 was added to each tube, and samples were bead-beaten as follows: three cycles of 1600 rpm for 120 s with two minutes incubation on ice between each cycle (FastPrep-96™) followed by 10 min of centrifugation (3486 × *g*). Afterwards 300 µL of supernatant was transferred to an S-block containing 300 µL of CD2 solution and 100 µL of nuclease-free water per well. The remaining steps were performed using the QIAcube HT kit (QIAGEN, Copenhagen, Denmark). DNA concentrations were measured using a Qubit dsDNA HS kit (Thermo Fisher Scientific, Waltham, MA) on an Infinite 200 Pro [25].

### 16S rRNA V4 amplicon library preparation and sequencing

Extracted DNA was quantified using Qubit HS assay kits (Thermo Fisher Scientific, Waltham, MA), and gDNA concentrations were measured using a Cytation 3 instrument (Agilent, Santa Clara, CA). Genomic DNA from Microbial Mock Community B v5.1L for 16S rRNA Gene Sequencing (BEI, Manassas, VA) was used as a positive control. DNA concentrations were normalized to 5 ng/µL using Qiagility Liquid Handler if above threshold, and 10 ng per sample was used as template for 16S rRNA amplification using TaKaRa Ex Taq^®^ DNA Polymerase Hot-Start Version and 515F/806R primers using a 30-cycle amplification. Amplicons were quantified as described above using a Qubit HS Assay kit and a Cytation 3 instrument. Library preparation underwent AMPure XP bead (Beckman, Indianapolis, IN) cleanup prior to quantification using the Qubit Broad Range kit (Thermo Fisher, MA), diluted to 2 nM, and denatured. The library was combined with the PhiX control at equal molarity and diluted to 1.5 pM for sequencing on an Illumina Miseq 600 cycle. Forward and reverse paired-end reads were demultiplexed using the QIIME1 v1.9.1 [26]. Reads were filtered for low-complexity poly-G sequences using the bbTools entropy filter at the level of 0.2. Divisive amplicon denoising algorithm 2 (DADA2) [27] in R (v4.2.2) was used for data processing, including quality filtering with a maximum expected error of 2, chimera removal, and taxonomic assignment to amplicon sequence variants (ASVs) using the SILVA v132 database [28]. Downstream filtering included removing low-frequent ASVs (<0.001%) and filtering potential background signals arising from reagents. ASVs present in >15% of negative controls and <15% of samples were removed outright. The mean of the remaining counts in ASVs was subtracted from that of the samples.

### Statistical analysis

#### Clinical data

Statistical analyses were conducted using R (v4.2.2) (R Foundation for Statistical Computing, Vienna, Austria) and GraphPad (v9.5.0, copyright licensed) (GraphPad Software, La Jolla, CA). Normally distributed parametric data were tested using Student’s *t*-test or one-way ANOVA. Non-normally distributed data were analysed using the Mann–Whitney (MW) or Kruskal–Wallis (KW) tests. Categorical data were analysed using the Chi-square or Fisher’s exact test when applicable. Outcome variables (EIM, number of EIMs) were analysed as whole (IBD) and disease-specific (CD and UC separately).

#### 16S rRNA analysis

Diversity indices were calculated using ‘phyloseq’ in R (v1.40.0) and ‘vegan’ (v2.6.4) [29,30] and compared between groups using the MW or KW test. Using ‘phyloseq’ distance matrices, unweighted UniFrac and weighted UniFrac were calculated and related to clinical variables using permutational analysis of variance test (PERMANOVA) via adonis2 of the ‘vegan’ package (v2.6.4) [30]. ASVs present in <10% of samples were removed prior to determining differentially abundant ASVs using a custom script that employs multiple statistical models (linear model, Poisson, negative binomial and zero-inflated negative binomial) and compared using the AIC before reporting the final estimate and *p* value (https://github.com/lynchlab-ucsf/lab-code/blob/master/SigTaxa/ManyModelScript.R) to each ASV dataset. False discovery rate corrections were made using the Benjamini–Hochberg method, and *p* values <.05 were considered significant. Only significant ASVs with a log difference >1 were included.

Through a data reduction approach, co-associated ASV modules were identified using sparse correlations for compositional data (SparCC) [31] using the SpiecEasi package in R [32]. Only ASVs present in ≥10% of samples were included in this analysis, and a correlation coefficient ≥0.5 was used to define co-associated ASVs as we have previously described [33]. Logistic regression was used to test for associations between EIM and ASV modules.

## Results

### Clinical characteristics

A total of 156 patients with IBD were included in the study, of whom 25 patients with clinical symptoms of EIMs were excluded from further analysis because the EIM diagnosis had not yet been confirmed by a medical specialist (ophthalmologist, rheumatologist, dermatologist, etc.) at the time of the study. The final study population of 131 IBD patients consisted of 86 patients with EIMs (IBD-EIM), of whom 60 had CD (CD-EIM) and 26 had UC (UC-EIM). A total of *n* = 45 patients did not have EIMs (IBD-C) of whom 21 had CD (CD-C) and 24 had UC (UC-C). Study participant characteristics are shown in Table 1. There was no significant difference in age between IBD-EIM and IBD-C groups; however, there were significantly more females in IBD-EIM (MW; *p* = .03). Additionally, body mass index (BMI) was significantly higher in the IBD-EIM group than in the IBD-C group (MW; *p* = .02) which was primarily driven by the CD-EIM group (CD-EIM vs. CD-C, MW; *p* = .006; UC-EIM vs. UC-C, MW; *p* = .22). Furthermore, self-reported disease severity scores were significantly increased in patients with EIMs, irrespective of their underlying clinical subtype (HBI: CD-EIM vs. CD-C, *χ*^2^; *p* = .02; SCCAI: UC-EIM vs. UC-C, FE; *p* = .02) together with increased Short Health Scale (SHS) scores reflective of lower quality of life. Montreal classification parameters such as age at diagnosis (*A*), location (*L*) and behaviour (*B*) categories did not differ between CD groups (*χ*^2^; *A*: *p* = .19, *L*: *p* = .76, *B*: *p* = .35); however, reflective of more severe disease, UC-EIM patients had a significantly higher prevalence of pancolitis compared to UC-C (extent of UC; *χ*^2^, *p* = .03). Treatment also varied between groups, with higher use of biologics in the IBD-EIM versus IBD-C group (biologics; FE; *p* < .01). The IBD-EIM group also exhibited a higher incidence of bone mineral disease (osteopenia/osteoporosis) (*χ*^2^; *p* = .009, Supplemental Table 1). EIMs, including the number of EIMs within the study group, are shown in Table 2.

**Table 1.** Study participant characteristics.

	IBD (*n* = 131)		CD (*n* = 81)		UC (*n* = 50)	
	IBD-EIM (*n* = 86)	IBD-C (*n* = 45)	CD-EIM (*n* = 60)	CD-C (*n* = 21)	UC-EIM (*n* = 26)	UC-C (*n* = 24)
Female (*n*/total (%))*	52/86 (60)*	18/45 (40)*	36/60 (60)	9/21 (43)	16/26 (62)	9/24 (38)
Age (mean (SD))	48 (±13)	44 (±14)	48 (±14)	44 (±13)	48 (±11)	44 (±14)
BMI (kg/m^2^) (mean (SD))*	28.1 (±5.3)*	25.6 (±5.3)*	28.4 (±5.4)*	24.8 (±3.9)*	27.4 (±5.2)	26.4 (±6.3)
BMI (*n*/total (%))*	*	*	*	*		
Underweight (<18.5)	0/86 (0)	1/45 (2)	0/60 (0)	1/21 (5)	0/26 (0)	0/24 (0)
Normal (18.5–24.9)	26/86 (30)	27/45 (60)	17/60 (28)	12/21 (57)	15/26 (58)	9/24 (38)
Overweight (25–29.9)	35/86 (41)	11/45 (25)	24/60 (40)	6/21 (29)	5/26 (19)	11/24 (46)
Obese (>30)	25/86 (29)	6/45 (13)	19/60 (32)	2/21 (9)	6/26 (23)	4/24 (16)
Years since IBD diagnosis (mean (SD))	15 (±9)	13 (±8)	16 (±9)	12 (±6)	14 (±8)	14 (±9)
Family disposition to IBD (*n*/total (%))	28/84 (33)	14/29 (33)	34/58 (40)	4/20 (20)	5/26 (20)	10/23 (43)
IBD related hospitalization (*n*/total (%))*	65/85 (76)*	20/45 (44)*	49/59 (83)*	11/21 (52)*	16/26 (62)	9/24 (38)
IBD related surgery (*n*/total (%))	32/86 (37)	12/45 (27)	29/60 (48)	12/21 (57)	3/26 (12)	0/24 (0)
*Montreal classification*						
A: age of diagnosis (*n*/total (%))						
A1: below 16 years	5/86 (6)	6/45 (13)	5/60 (8)	1/21 (5)	0/26 (0)	5/24 (21)
A2: between 17 and 40 years	67/86 (78)	29/45 (65)	48/60 (80)	14/21 (66)	19/26 (73)	15/24 (63)
A3: above 40 years	14/86 (16)	10/45 (22)	7/60 (12)	6/21 (29)	7/26 (27)	4/24 (16)
L: disease location in CD (*n*/total (%))						
L1: ileal			7/58 (12)	2/21 (10)		
L2: colonic			28/58 (48)	12/21 (57)		
L3: ileocolonic			14/58 (26)	3/21 (14)		
L4: upper GI involvement			9/58 (16)	4/21 (19)		
B: behaviour in CD (*n*/total (%))						
B1: non-stricturing, non-penetrating			47/58 (81)	14/21 (67)		
B2: stricturing			6/58 (10)	3/21 (14)		
B3: penetrating			5/58 (9)	4/21 (19)		
E: disease extent in UC (*n* (%))*					*	*
E1: ulcerative proctitis					3/26 (11)	6/24 (25)
E2: left sided colitis					7/26 (27)	12/24 (50)
E3: extensive colitis					16/26 (62)	6/24 (25)
*Treatment*						
No treatment (*n*/total (%))	5/86 (6)	6/45 (13)	3/60 (5)	4/21 (19)	2/26 (8)	2/24 (8)
Standard treatment (*n*/total (%))*	37/86 (43)*	29 (64)*	17/60 (28)	9/21 (43)	20/26 (77)	20 (83)
5-ASA (topical/systemic)*	22/86 (26)*	20/45 (44)*	4/60 (7)	0/21 (0)	18/26 (70)	19/24 (79)
Corticosteroids (topical/systemic)	3/60 (5)	1/45 (2)	2/60 (3)	0/21 (0)	1/26 (4)	1/24 (4)
Azathioprine*	11/75 (13)*	24/45 (29)*	10/60 (17)	7/21 (33)	1/26 (4)*	6/24 (24)*
Mercaptopurine	2/86 (2)	1/45 (2)	1/60 (2)	1/21 (5)	1/26 (4)	1/24 (7)
Methotrexate	5/86 (6)	0/45 (0)	2/60 (3)	0/21 (0)	3/26 (12)	0/24 (0)
Biologics and targeted small molecule drugs (*n*/total (%))*	70/86 (81)*	23/45 (51)*	56/60 (93)	14/21 (67)	14/26 (54)	9/24 (38)
TNF-α inhibitors*	55/86 (64)	26/45 (58)	41/60 (68)	13/21 (62)	14/26 (54)*	6/24 (25)*
Vedolizumab	11/86 (13)	2/45 (4)	11/60 (18)	1/21 (5)	1/26 (4)	0/24 (0)
Ustekinumab	4/86 (5)	0/45 (0)	4/60 (7)	0/21 (0)	0/26 (0)	0/24 (0)
Other	2/86 (2)	0/45 (0)	1/60 (2)	0/21 (0)	0/26 (0)	1/24 (4)
*Quality of life/disease activity*						
Short Health Score*	144 (±92)*	68 (±73)*	153 (±95)*	77 (±96)*	123 (±85)*	60 (±44)*
Harvey–Bradshaw Index (HBI)*			*	*		
Remission (<5)			30/59 (51)	17/21 (81)		
Mild activity (5–7)			15/59 (25)	4/21 (19)		
Moderate activity (8–16)			14/59 (24)	0/21 (0)		
Severe activity (>16)			0/59 (0)	0/21 (0)		
Simple Clinical Colitis Activity Index (SCCAI)						
Remission (≤2)					16/26 (62)	18/24 (75)
Mild (3–5)					7/26 (27)	6/24 (25)
Moderate (6–11)					4/26 (15)	0/24 (0)
Severe (≥12)					0/26 (0)	0/24 (0)

* *p* Value < .05. TNF-α inhibitors: Tumor Necrosis Factor alpha inhibitor; 5-ASA: 5-aminosalicylic acid.

**Table 2.** Extraintestinal manifestations.

	EIM (*n* = 86)	CD-EIM (*n* = 60)	UC-EIM (*n* = 26)
Number of EIMs			
1	60	39	21
2	22	18	4
≥3	4	2	1
EIM at incl.	70	49	21
Types of EIMs			
Arthropathies	64	45	19
Spondyloarthritis (SpA)	34	27	7
Arthropathy	30	18	12
Ocular manifestations	25	19	6
Uveitis*	19	15	4
Other	6	4	2
Hepatobiliary manifestations	14	6	8
PSC	10	4	6
Autoimmune hepatitis	4	2	2
Skin manifestations	12	12	0
Erythema nodosum	12	12	0
Other	28	26	2

*Incl. anterior uveitis, iridocyclitis, posterior uveitis.

### Immunological characteristics

**Figure 1.**
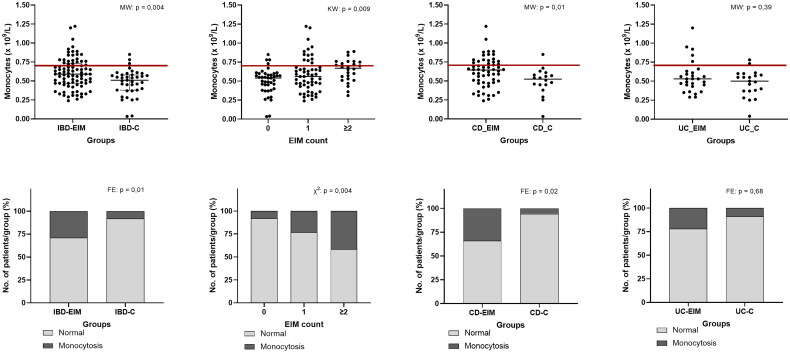
Blood monocyte concentration is higher in IBD patients with EIMs. **(**A) Monocyte concentration (×10^9^/L) between IBD-EIM and IBD-C. Values above the redline are above reference levels and represents monocytosis (>0.70 × 10^9^/L). (B) Monocyte concentration by EIM count (0, 1, ≥2). (C) Monocyte concentration between CD-EIM and CD-C. (D) Monocyte concentration between UC-EIM and UC-C. (E) Monocytosis in IBD-EIM vs. IBD-C. (F) Monocytosis by EIM count. (G) Monocytosis between CD-EIM and CD-C. (H) Monocytosis between UC-EIM and UC-C. MW: Mann–Whitney’s test; KW: Kruskal–Wallis test; FE: Fisher’s exact test. Monocytes were not available for all participants, leading to reduced group sizes for this analysis: IBD-EIM (*n* = 85), IBD-C (*n* = 40), CD-EIM (*n* = 59), CD-C (*n* = 18), UC-EIM (*n* = 26) and UC-C (*n* = 22). *p* Values < .05 were considered significant.

Standard biomarkers for disease activity were measured for each participant (Supplemental Table 2) and between-group comparisons were made. Mean f-calprotectin in both IBD-EIM and IBD-C was above the IBD reference levels (>200 mg/kg), and significantly higher f-calprotectin was observed in IBD-EIM (MW; *p* = .04). CRP and leukocytes differed between IBD-EIM and IBD-C (CRP: MW; *p* = .006, leukocytes: MW; *p* = .003), although means of both remained within reference level (CRP; <8 mg/L, leukocyte; 3.5–8.8 × 10^9^/L). Difference in leukocytes was driven by increase in IBD-EIM of specific leukocyte subtypes: monocytes (MW; *p* = .003, Figure 1(A)) and, to a lesser extent, neutrophils (MW; *p* = .02) and lymphocytes (MW; *p* = .02). The prevalence of monocytosis (>0.70 × 10^9^/L) was higher in the IBD-EIM group (FE; *p* = .01, Figure 1(E)), and increased with the number of EIMs (FE; *p* = .005, Figure 1(F)), as did overall monocyte concentration (linear regression: *R*^2^ = 0.07, *p* = .004, KW: *p* = .009, Figure 1(B)). When analysing CD and UC separately, CD-EIM had both higher monocyte concentration (MW; *p* = .01, Figure 1(C,D)) as well as monocytosis compared to CD-C (FE; *p* = .03, Figure 1(G)). However, no significant difference was observed between the UC-EIM and UC-C groups. Neutrophils and lymphocytes were also significantly elevated in the IBD-EIM versus IBD-C group (MW; *p* = .01). However, mean neutrophils and lymphocyte concentration remained within reference level (neutrophils: 2.0–7.0 × 10^9^/L, lymphocytes; 1.3–3.5 × 10^9^/L) and the prevalence of neutrophilia (>7.0 × 10^9^/L) and lymphocytosis (>3.5 × 10^9^/L) was not significantly different between IBD-EIM and IBD-C. Because both UC-EIM and CD-EIM groups exhibited significantly higher disease activity compared to the control groups, disease activity was investigated as a confounding factor related to monocyte concentration. Monocyte concentration and disease activity were not significantly correlated in CD or UC; however, monocyte concentration and f-calprotectin showed a significant but low correlation (*R*^2^ = 0.04, *p* = .03, Supplemental Figure 1). Lastly, IBD-EIM was characterized by lower albumin levels (*p* = .0003) which was also related to the number of EIMs experienced by patients (*p* = .0009), however, cases of hypoalbuminaemia did not differ between groups.

### Fecal microbiota diversity relates to EIM

**Figure 2.**
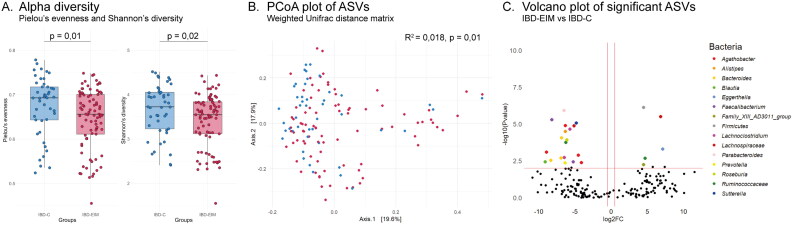
Fecal microbiota amplicon sequence variants (ASVs) differ between inflammatory bowel diseases patients with (IBD-EIM) or without (IBD-C) extraintestinal manifestations (EIM). (A) Alpha-diversity indices between IBD-EIM (red) and IBD-C (blue) (Pielou’s evenness, *p* = .01, Shannon’s diversity, *p* = .02). (B) Principal coordinate analysis plot depicts beta-diversity distance matrices between IBD-EIM (red) and IBD-C (blue) patients (weighted UniFrac, PERMANOVA; *R*^2^ = 0.02, *p* = .01). (C) Volcano plot of significant ASVs between IBD-EIM and IBD-C. Significant ASVs (*p*.fdr < .05, LogFC > 1) are coloured by genus or the highest taxonomic level identified. Significantly enriched ASVs in IBD-EIM (*n* = 5) are depicted on the right axis and ASVs depleted in IBD-EIM (*n* = 19) are depicted on the left axis.

A total of 3362 different bacterial ASVs were identified representing 11 different phyla, 19 classes, 67 families, 196 genera and 303 species. The mean observed number of ASVs per participant was 210 for IBD-EIM (*n* = 86) and 240 for IBD-C (*n* = 45), which was trending towards significance (*t*-test; *p* = .08). Additional bacterial diversity measures also differed between groups. IBD-EIM was characterized by a significantly lower fecal microbiota diversity (Shannon diversity; MW test, *p* = .02, Figure 2(A)) and evenness (Pielou’s evenness; MW test, *p* = .01; Figure 2(A)).

### Fecal microbiota composition relates to EIM

The fecal microbiota composition was compared between participants (beta-diversity) and IBD-EIM and IBD-C was found to be compositionally distinct (PERMANOVA; weighted UniFrac, *R*^2^ = 0.018, *p* < .01, Figure 2(B); Supplemental Table 3). The variance in fecal microbiota composition between participants was, as expected, related mostly to the dominant bacterial family and genus present (PERMANOVA; weighted UniFrac, genus: *R*^2^ = 0.039, *p* < .001, family: *R*^2^ = 0.024, *p* < .001). Other clinical variables identified as significantly explaining microbiota variance, included disease subtype (CD/UC; PERMANOVA; weighted UniFrac, *R*^2^ = 0.07, *p* < .001), and IBD surgery (yes/no; PERMANOVA; weighted UniFrac, *R*^2^ = 0.04, *p* < .001). All variables with significant impact on microbiota variance are listed in Supplemental Table 3. Several clinical variables identified as significant between IBD-EIM and IBD-C were not found to be related to overall variance in gut microbiota composition, including BMI, treatment and F-calprotectin. However, albumin and leukocytes did explain a significant variance with a trend towards significance for monocytes and neutrophils in some beta diversity distance matrices (Supplemental Table 3).

### Bacterial taxa related to EIM

Differentially abundant ASVs were identified using a custom multi-model (CMM) script (to account for variance in data distributions across ASVs). Following adjustment for clinical variables previously found to be related to variance in microbiota composition (e.g. disease subtype, IBD surgery), twenty-four ASVs were found to differ significantly in relative abundance between the IBD-EIM and IBD-C groups (*p*.fdr < .05; Figure 2(C)). Fecal microbiota of IBD-EIM patients was relatively enriched for five ASVs and relatively decreased for 19 ASVs. The relatively enriched ASVs included *Eggerthella lenta*, an unspecified species of *Family XIII AD3011 group genus* and three unspecified members of *Lachnospiraceae*, *Ruminococcaceae* and *Firmicutes*, respectively. The 19 relatively decreased ASVs in IBD-EIM included members of the following genera *Agathobacter*, *Alistipes*, *Bacteroides*, *Blautia*, *Faecalibacterium*, *Lachnoclostridium*, *Roseburia* and *Sutterella* amongst others listed in Table 3. Subanalysis for CD yielded five significantly different ASVs between CD-EIM and CD-C after adjusting for disease severity (HBI), IBD surgery and Montreal location (Supplemental Table 4). Subanalysis of UC yielded 17 significant ASVs between UC-EIM and UC-C (Supplemental Table 5). Only three ASVs remained significant or trending towards significance in both UC and CD subanalyses (decreased *Bacteroides vulgaris* (ASV_48), *Bacteroides fragilis/koreensis/kribbi/ovatus* (ASV 204) and increased *Lachnospiraceae NA* (ASV_93)). Additionally, ASVs belonging to *Agathobacter* remained significantly decreased in the subanalysis for CD (ASV_36) and UC (ASV_158), as did ASVs annotated as unspecified *Lachnospiraceae* for CD (ASV_184) and UC (ASV_271). For CD, an unspecified *Firmicute* (ASV 242) remained significantly increased and a *Dorea*, but none of the increased ASVs in UC subanalysis were present in IBD combined and included an ASVs belonging to *Subdoligranulum*, *Blautia* and unspecified *Lachnospiraceae.* Finally, several ASVs belonging to *Sutterella*, *Faecalibacterium* and *Lachnoclostridium* amongst others, remained significantly decreased in UC (Supplemental Table 5).

**Table 3.** Significant ASVs between IBD-EIM and IBD-C.

ASV	*p*.fdr	Log2 FC	Mean diff.	Phylum	Family	Genus	Species
ASV_20	.0344	−9.14	−563	*Firmicutes*	*Lachnospiraceae*	*Blautia*	*obeum*
ASV_36	.0112	−8.94	−490	*Firmicutes*	*Lachnospiraceae*	*Agathobacter*	*NA*
ASV_48	.0292	−8.34	−324	*Bacteroidota*	*Bacteroidaceae*	*Bacteroides*	*vulgatus*
ASV_91	.0002	−8.21	−296	*Firmicutes*	*Ruminococcaceae*	*Faecalibacterium*	*NA*
ASV_94	.0221	−6.94	−123	*Bacteroidota*	*Bacteroidaceae*	*Bacteroides*	*uniformis*
ASV_204	.0016	−6.87	−117	*Bacteroidota*	*Bacteroidaceae*	*Bacteroides*	*fragilis/* *koreensis/* *kribbi/ovatus*
ASV_241	.0001	−6.52	−92	*Bacteroidota*	*Tannerellaceae*	*Parabacteroides*	*distasonis*
ASV_233	.0221	−6.49	−90	*Firmicutes*	*Lachnospiraceae*	*Lachnoclostridium*	*NA*
ASV_50	.0368	−6.37	−83	*Bacteroidota*	*Prevotellaceae*	*Prevotella*	*copri*
ASV_158	.0004	−6.33	−81	*Firmicutes*	*Lachnospiraceae*	*Agathobacter*	*NA*
ASV_108	.0007	−6.33	−80	*Bacteroidota*	*Rikenellaceae*	*Alistipes*	*putredinis*
ASV_272	.0028	−6.28	−78	*Firmicutes*	*Ruminococcaceae*	*NA*	*NA*
ASV_192	.0020	−6.11	−69	*Firmicutes*	*Lachnospiraceae*	*Roseburia*	*NA*
ASV_307	.0005	−5.70	−52	*Firmicutes*	*Lachnospiraceae*	*Lachnoclostridium*	*edouardi*
ASV_202	.0344	−5.26	−38	*Firmicutes*	*Ruminococcaceae*	*Faecalibacterium*	*NA*
ASV_271	.0004	−5.13	−35	*Firmicutes*	*Lachnospiraceae*	*NA*	*NA*
ASV_342	.0003	−4.82	−28	*Proteobacteria*	*Sutterellaceae*	*Sutterella*	*massiliensis/* *stercoricanis/* *wadsworthensis*
ASV_284	.0170	−4.54	−23	*Firmicutes*	*Lachnospiraceae*	*Agathobacter*	*NA*
ASV_267	.0368	−4.13	−18	*Firmicutes*	*Lachnospiraceae*	*Agathobacter*	*NA*
ASV_242	.0001	4.45	22	*Firmicutes*	*NA*	*NA*	*NA*
ASV_217	.0450	4.46	22	*Firmicutes*	*Anaerovoracaceae*	*Family_XIII_AD3011_group*	*NA*
ASV_296	.0221	4.67	25	*Firmicutes*	*Ruminococcaceae*	*NA*	*NA*
ASV_93	.0002	6.84	115	*Firmicutes*	*Lachnospiraceae*	*NA*	*NA*
ASV_83	.0074	7.10	137	*Actinobacteriota*	*Eggerthellaceae*	*Eggerthella*	*lenta*

### Bacterial networks associated with EIMs

**Figure 3.**
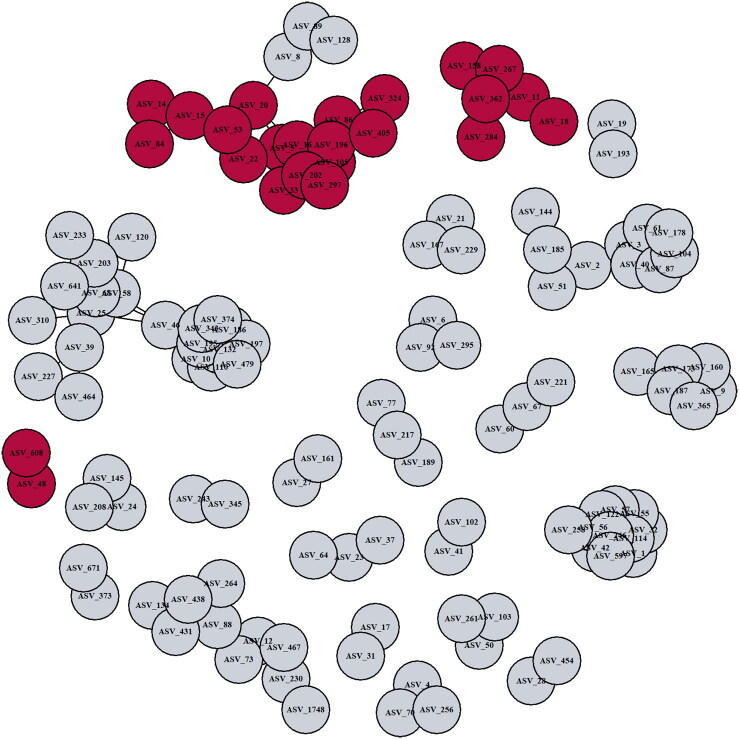
Fecal microbiota bacterial networks differ between IBD-EIM and IBD-C. Twenty-seven bacterial networks were identified using sparse correlations for compositional data (SparCC) on amplicon sequence variants (ASVs) with a correlation cutoff of 0.5. Three bacterial networks (module 7 (*n* = 16), module 8 (*n* = 6) and module 23 (*n* = 2)) were significantly different between IBD-EIM and IBD-C (logistic regression, *p* < .05), and all were negatively correlated with IBD-EIM. Significant modules are marked in dark red, and non-significant modules are marked in grey.

A data reduction approach using SparCC was conducted to identify bacterial co-association modules related to EIM. Twenty-five bacterial networks of ASVs with a correlation ≥0.5 were identified. Of these, three networks were significantly related to EIM (logistic regression, *p* < .05, Figure 3), and all three were depleted in IBD-EIM fecal microbiomes. Module 7 (*n* = 16, *p* = .004) consists of several *Faecalibacterium* ASVs, *Blautia* and *Erysipelotrichaceae_UCG-003* amongst others. Module 8 (*n* = 6, *p* = .008) primarily consisting of *Agathobacter* and module 23 (*n* = 2, *p* = .025) consisting of *Bacteroides* were also negatively associated with EIM. All three modules contained some of the same ASVs, which were also significantly decreased in IBD-EIM in our comparative analysis (ASV_20; *Blautia obeum*, ASV_202; *Faecalibacterium NA*, ASV_158; *Agathobacter NA*, ASV_48; *Bacteroides vulgaris*) (Table 4).

**Table 4.** Significant bacterial network modules with correlation ≥0.5.

Module	Module size	*p* Value	ASV members
Module 7	16	.004	*Blautia obeum* *Blautia faecis* *Dorea longicatena* *Erysipelotrichaceae_UCG-003 bacterium* *Erysipelotrichaceae_UCG-003 NA* *Faecalibacterium cf./prausnitzii* *Faecalibacterium prausnitzii* *Faecalibacterium prausnitzii* *Faecalibacterium NA* *Faecalibacterium NA* *Faecalibacterium NA* *Faecalibacterium NA* *Faecalibacterium NA* *Monoglobus NA* *Ruminococcaceae NA* *Ruminococcaceae NA*
Module 8	6	.008	*Agathobacter NA* *Agathobacter NA* *Agathobacter NA* *Agathobacter NA* *Agathobacter NA* *Fusicatenibacter saccharivorans*
Module 25	2	.025	*Bacteroides vulgatus* *Bacteroides NA*

## Discussion

In this study, we found that IBD patients with EIMs exhibited increased self-reported disease severity score together with lower quality of life, higher BMI, higher use of biological treatment and more cases of bone mineral disease. These clinical features of more severe disease were bolstered by immunological markers, including elevated f-calprotectin concentrations and increased CRP and leukocytes in those with EIMs, which latter cellular features appear to be largely due to the CD patient population. The fecal microbiota of patients with IBD and EIMs differs from that of patients without such clinical manifestations. Differences were apparent in several microbiota measures, such as diversity and composition, with loss of health-associated gut bacteria and enrichment of bacteria previously implicated in immune-mediated diseases.

Of these, *Eggerthella lenta* was the most enriched taxon in the IBD-EIM group. *Eggerthella* has previously been linked to rheumatoid arthritis [34] and IBD [35]. A recent study found T-lymphocyte subtype Th17 activating properties in *Eggerthella lenta* [35]. Th17 activation was achieved by lifting inhibition of Th17 transcription factor Rorγt. *E. lenta* strains had varying Th17 activation capabilities, which were attributed to the cardiac glycoside reductase 2 (cgr2) enzyme [35]. Cgr2 was able to induce IL-17a production. Lastly, cgr2+ *E. lenta* strains were found to deplete steroidal glycoside compounds, which have been described to be decreased in IBD patients with higher disease severity [35].

*Agathobacter*, a genus classified in 2016 [36], was significantly depleted in IBD-EIM in both relative differential abundance, but also as the dominant member of a significantly depleted bacterial network (module 8). Additionally, disease-specific subanalyses revealed decreased abundance of ASVs annotated as *Agathobacter*, which was evident in both CD-EIM and UC-EIM. *Agathobacter* is a member of the *Lachnospiraceae* family and includes the former *Eubacterium rectale*, which is now referred to as *Agathobacter rectalis* [36]. In line with our results, *Agathobacter* has previously been described as decreased in UC [37,38] and CD [39] compared with healthy controls [40]. Additionally, decreased *Agathobacter* abundance has been described in other conditions, including PSC [41], neoplasia in UC [42], colorectal cancer [43] and systemic lupus erythematosus [44]. *A. rectalis* is known for its ability to produce butyrate [36], an important metabolite in the human gut that serves as the primary energy source for intestinal epithelial cells [45]. Notably, a recent study identified *A. rectalis* as one of the highest producers of butyric acid amongst 110 *Lachnospiraceae* strains [45].

The most significantly depleted ASV was annotated as *Blautia*, also a member of the *Lachnospiraceae* family*. Blautia* is recognized as an important member of the human gut microbiota. To date, 20 different species have been discovered and these exhibit significant genetic variability, potentially resulting in diverse functional capacities [46]. *Blautia* has the capacity to produce secondary metabolites, such as bacteriocins; thus, it is speculated that *Blautia* has an important function in inhibiting colonization of pathogenic bacteria and ultimately influencing gut microbiota composition [46]*. Blautia* abundance in IBD is conflicting, as decreased abundance has been reported for CD, specifically decreased in inflamed sites compared to non-inflamed sites [47], and increased abundance has been reported for UC [48]. However, both studies were based on 16S rRNA sequencing and could not provide species- or strain-resolved information on these genera. Additionally, the study examined mucosal samples and not stool samples. Interestingly, stool samples from CD patients undergoing a flare and *Clostridioides difficile* infection showed a significant depletion of *Blautia* compared to CD patients in remission [49]. *Blautia* depletion has been previously described in stool samples from patients with uveitis [19, 50] and patients with SpA [19]; however, enriched abundance has been reported for psoriasis [51]. Caution has been advised towards making general conclusions on *Blautia*’s impact on human health and disease, as different species could exert different effects [46], emphasizing the need for in-depth interspecies research.

*Bacteroides vulgatus* was also decreased in IBD-EIM. *B. vulgatus* has been found to reduce acute inflammation and intestinal injury in mice after lipopolysaccharide (LPS) challenge, mainly through modulation of cytokine production and gut microbiota composition [52], which has also been described in other mouse models [53,54]. Conversely, increased *B. vulgatus* has been identified in adenomatous polyps and colon cancer patients compared to healthy subjects [55] and has been implicated in UC disease activity [56].

To account for the intricate nature of the gut microbiota, which functions as a complex community, we employed a reductionist approach to identify modules of co-associated bacteria, and subsequently tested whether these were significantly different between IBD-EIM and IBD-C. The largest module (*n* = 17) primarily consisted of *Firmicutes* members, previously described as decreased in IBD patients compared to healthy subjects. Several of the significantly differentially abundant taxa were also identified in the significant modules, indicating that these taxa together form a functional unit, which could be protective of EIM development. Additional studies determining co-associated functional gene and metabolite modules associated with IBD-EIM are warranted to fully understand which microbial functions protect against EIM development.

Monocyte count and cases of monocytosis were greater in IBD-EIM than in IBD-C. A recent study found elevated monocyte counts in one-third of IBD patients within a 6-year period, with monocytosis correlated to greater disease activity and worsened clinical outcomes, hospitalization and surgery [57]. The IBD-EIM group had higher disease severity and activity measures (HBI, SCCAI and f-calprotectin levels) than the IBD-C group, which could explain the difference between groups; however, linear regression did not relate monocyte concentration to HBI and SCCAI, however, a correlation was found for f-calprotectin and monocytes. Some EIMs typically occur during IBD flares, whereas others occur independently of flares; both scenarios are represented in the current study group. To our knowledge, this is the first study describing a relationship between monocytosis and EIMs, which may implicate the innate immune system in EIM development.

It is important to recognize and address the limitations of this study. First, the inherent heterogeneity within the study population, with varying disease activity, location of disease, history of IBD related surgery, and treatments, poses a methodological challenge which can complicate comparisons. Consequently, steps were applied to identify confounding factors within the dataset. A large set of clinical variables was investigated for differences between the groups and their influence on gut microbiota composition. Certain clinical variables differed between the IBD-EIM and IBD-C groups, which could impact the difference in microbiota observed between groups, of which BMI was one of the significant variables. BMI is recognized as an important factor related to gut microbiota composition [58]. Overweight (BMI: 25–30 kg/m^2^) and obese (BMI >30 kg/m^2^) IBD patients have been described as having a higher incidence of EIMs than normal (BMI: 20–25 kg/m^2^) and underweight (BMI: <20 kg/m^2^) patients, which persisted even after removing arthralgia from the analysis, a prevalent symptom related to obesity [58]. However, BMI was not found to be a significant factor accounting for the microbial composition in this IBD study population. In contrast, disease severity was significantly different between IBD-EIM and IBD-C groups and contributed significantly to gut microbial composition in the overall study population. Thus, this variable was adjusted for in the downstream analysis, together with other significant variables (Supplemental Table 3).

Second, the identification of more cases of bone mineral disease in IBD-EIM could be a result of more steroid use within this group and thus increased frequency of dual-energy X-ray absorptiometry (DXA) scans; however, these data were not available for the study participants. More research is needed to determine whether EIM and bone mineral disease are associated.

Third, while our investigation focuses on the history of EIMs and the number of EIMs and provides new insight into the potential role of gut microbiota in EIMs, further research is needed. Specifically, subpopulations with current EIMs or specific EIM type analyses could unveil additional nuanced microbiota patterns and provide valuable insights. Fourth, 16S rRNA analysis only provides confident resolution to genus level, missing potential differences at species and strain levels. Other omic-analyses, such as metagenomics, are needed to further investigate a possible microbial link to EIMs in IBD. Lastly, the current study focused exclusively on gut bacteria; however, for a comprehensive understanding of the potential role of different types of microorganisms in EIMs, it is imperative to investigate the whole inter-kingdom community, including the mycobiome, virome and archaeome.

## Conclusions

Fecal microbiota of IBD patients with EIMs is distinct from that of IBD patients without EIMs. The fecal microbiota of IBD patients with EIMs is characterized by lower alpha diversity and has a distinct composition that lacks important short-chain fatty acid-producing bacteria from the *Lachnospiraceae* family, such as *Agathobacter* and *Blautia*. Additionally, *Eggerthella lenta*, a species recently implicated in Th17 activation in IBD, was increased in patients with IBD-EIM. This study’s inclusion of a large and heterogeneous IBD population indicates that relationships between the gut microbiota and IBD-EIMs are evident and warrant further investigation to uncover specific microbiome functional traits associated with EIMs in this patient population.

## Supplementary Material

Supplemental Material

## Data Availability

The data supporting the findings of this study are available from the corresponding author [S.H.] upon reasonable request.
