# Supplementary material for: Fecal microbiota is associated with extraintestinal manifestations in inflammatory bowel disease
Source: Ann Med. 2024 Apr 22;56(1):2338244. doi: 10.1080/07853890.2024.2338244 (PMC11036898; doi:10.1080/07853890.2024.2338244)
Supplement: Supplemental Material [file IANN_A_2338244_SM5386.docx]

# **Supplemental Tables**

## **Supplemental table 1**

| Comorbidities | IBD-EIM  (n = 86) | IBD-C  (n = 45) | CD EIM  (n = 60) | CD C  (n = 21) | UC_EIM  (n = 26) | UC_C  (n=24) |
| --- | --- | --- | --- | --- | --- | --- |
| Affected Bone Mineral Density*  *Osteopenia/osteoporosis* | 33/86*  (39%) | 8/45*  (18%) | 25/60  (42%) | 4/21  (19%) | 8/26  (31%) | 4/24  (17%) |
| Thrombo-embolic disease | 7/86  (8%) | 0/45  (0%) | 7/60  (12%) | 0/21  (0%) | 0/26  (0%) | 0/24  (0%) |
| Cholelithiasis | 18/86  (21%) | 4/45  (9%) | 11/60  (18%) | 2/21  (10%) | 7/26  (27%) | 2/24  (8%) |
| Urolithiasis | 12/86  (14%) | 3/45  (7%) | 10/60  (17%) | 1/21  (5%) | 2/29  (8%) | 2/23  (8%) |
| Atopy | 28/86  (33%) | 11/45  (24%) | 19/60  (32%) | 4/21  (21%) | 9/26  (35%) | 7/24  (30%) |
| Asthma | 11/86  (13%) | 1/45  (2%) | 10/60  (17%) | 0/21  (0%) | 1/26  (4%) | 1/24  (4%) |
| Psoriasis | 17/86  (20%) | 4/45  (9%) | 15/60  (17%) | 2/21  (10%) | 2/26  (8%) | 2/24  (8%) |
| Psychiatric disease | 9/86  (10%) | 4/45  (9%) | 8/59  (13%) | 2/21  (10%) | 1/26  (4%) | 2/24  (8%) |

Atopy: One or more of the following: Allergic rhinitis. eczema and/or asthma

*Significant. p ≤ 0.05

## **Supplemental table 2**

|  | Reference  Interval | IBD-EIM  (n = 86) | IBD-C  (n = 45) | CD EIM  (n = 60) | CD C  (n = 21) | UC_EIM  (n = 26) | UC_C  (n=24) |
| --- | --- | --- | --- | --- | --- | --- | --- |
| F-calprotectin* | <200 mg/kg | 343(±519)* | 205(±363)* | 323(±512) | 223(±320) | 390(±542)* | 187(±406)* |
| CRP* | <8 mg/L | 4.9(±6.5)* | 4.0(±8.4)* | 5.4(±7.3) | 4.5(±9.1) | 3.7(±4.3) | 3.6(±8.0) |
| Leukocytes | 3.5-10x10^9^/L | 7.5(±2.1)* | 6.4(±2.2)* | 7.8(±2.1) | 7.0(±2.7) | 6.7(±1.8) | 5.9(±1.5) |
| *Neutrophils**  *Lymphocytes**  *Monocytes**  *Eosinophils*  *Basophils* | 2.0-7.0  1.3-3.5  0.2-0.7  < 0.5  < 0.1 | 4.2(±1.6)*  2.2 (±0.9)*  0.6 (±0.2)*  0.17(±0.13)  0.05(±0.02) | 3.7(±1.5)*  1.8(±0.8)*  0.5(±0.2)*  0.17(±0.14)  0.04(±0.02) | 4.7(±1.6)  2.3 (±0.9)  0.6 (±0.2)*  0.16(±0.12)  0.04(±0.02) | 4.1(±1.5)  2.0(±1.0)  0.5(±0.2)*  0.16(±0.14)  0.04(±0.02) | 3.8(±1.4)  2.1(±0.7)  0.6(±0.2)  0.16(±0.13)  0.05(±0.02) | 3.4(±1.5)  1.7(±0.6)  0.5(±0.2)  0.17(±0.13)  0.04(±0.02) |
| Hemoglobin | 7.3-10.5 mmol/L | 8.7(±0.8) | 8.7(±0.7) | 8.7(±0.8) | 8.6(±0.6) | 8.7(±0.9) | 8.8(±0.8) |
| Thrombocytes | 145-400x10^9^/L | 284(±76) | 269(±70) | 294(±77) | 288(±83) | 261(±72) | 252(±52) |
| ALAT* | 10-50U/L | 41(±55)* | 26(±15)* | 36(±53)* | 24(±13)* | 50(±57) | 28(±17) |
| Albumin* | 36-46g/L | 37(±4)* | 40(±4)* | 37(±4)* | 40(±5)* | 38(±3) | 40(±3) |
| Alkaline phosphatase* | 35-105U/L | 101(±98) | 66(±17) | 82(±48) | 71(±17) | 147(±156)* | 62(±16)* |
| Amylase | 10-65U/L | 28(±16) | 28(±15) | 28(±14) | 29(±16) | 26(±20) | 27(±15) |
| Bilirubin | 5-25μmol/L | 9.6(±5) | 10(±6) | 9.4(±5) | 10.2(±5) | 9.8 ±6) | 9.8 (±6) |

*Significant. p ≤ 0.05. all values are mean values (±standard deviations). hemoglobin and thrombocyte reference interval is a combination of female and male reference intervals.

## **Supplemental table 3**

| PERMANOVA results of Amplicon Sequence Variants (ASV) | | | | | | | | | | | | | |
| --- | --- | --- | --- | --- | --- | --- | --- | --- | --- | --- | --- | --- | --- |
| Analysis subset | **IBD (n = 131)** | | | | **CD (n = 81)** | | | | **UC (n = 50)** | | | | |
| UniFrac distances | **Unweighted** | | **Weighted** | | **Unweighted** | | **Weighted** | | **Unweighted** | | | **Weighted** | |
| Measures | **R^2^** | **p** | **R^2^** | **p** | **R^2^** | **p** | **R^2^** | **p** | **R^2^** | **p** | **R^2^** | | **p** |
| Dominant genus | 0.18 | 0.001 | 0.41 | 0.001 | 0.18 | 0.005 | 0.44 | 0.001 | 0.32 | 0.01 | 0.46 | | 0.001 |
| Dominant family | 0.11 | 0.001 | 0.25 | 0.001 | 0.10 | 0.01 | 0.25 | 0.001 | 0.19 | 0.001 | 0.33 | | 0.001 |
| Sex (female/male) | 0.01 | 0.12 | 0.01 | 0.22 | 0.01 | 0.32 | 0.02 | 0.17 | 0.02 | 0.29 | 0.03 | | 0.07 |
| Age | 0.007 | 0.55 | 0.008 | 0.36 | 0.01 | 0.68 | 0.01 | 0.47 | 0.03 | 0.04 | 0.02 | | 0.25 |
| BMI | 0.008 | 0.21 | 0.003 | 0.94 | 0.01 | 0.19 | 0.006 | 0.88 | 0.02 | 0.24 | 0.02 | | 0.33 |
| IBD related surgery (yes/no) | 0.07 | 0.001 | 0.06 | 0.001 | 0.08 | 0.001 | 0.05 | 0.002 | 0.04 | 0.02 | 0.06 | | 0.009 |
| IBD subtype (CD/UC) | 0.02 | 0.01 | 0.03 | 0.001 | - | - | - | - | - | - | - | | - |
| SHS | 0.03 | 0.001 | 0.03 | 0.001 | 0.04 | 0.004 | 0.04 | 0.006 | 0.02 | 0.54 | 0.01 | | 0.91 |
| HBI (CD only) | - | - | - | - | 0.07 | 0.001 | 0.06 | 0.001 | - | - | - | | - |
| L_location (CD only) | - | - | - | - | 0.08 | 0.001 | 0.09 | 0.001 | - | - | - | | - |
| SCCAI (UC only) | - | - | - | - | - | - | - | - | 0.02 | 0.48 | 0.008 | | 0.97 |
| E_extent (UC only) | - | - | - | - | - | - | - | - | 0.11 | 0.60 | 0.07 | | 0.99 |
| IBD admission | 0.04 | 0.001 | 0.05 | 0.003 | 0.07 | 0.001 | 0.06 | 0.003 | 0.01 | 0.77 | 0.01 | | 0.68 |
| Biological treatment (yes/no) | 0.02 | 0.009 | 0.01 | 0.09 | 0.01 | 0.80 | 0.009 | 0.72 | 0.04 | 0.007 | 0.02 | | 0.40 |
| Standard treatment | 0.01 | 0.08 | 0.01 | 0.06 | 0.008 | 0.95 | 0.02 | 0.13 | 0.04 | 0.01 | 0.02 | | 0.53 |
| EIM (yes/no) | 0.01 | 0.04 | 0.02 | 0.01 | 0.02 | 0.11 | 0.02 | 0.09 | 0.02 | 0.73 | 0.01 | | 0.68 |
| EIM count (0,1,≥2) | 0.01 | 0.04 | 0.02 | 0.01 | 0.03 | 0.18 | 0.02 | 0.08 | 0.03 | 0.67 | 0.03 | | 0.82 |
| Arthralgia at incl (yes/no) | 0.02 | 0.06 | 0.03 | 0.02 | 0.03 | 0.21 | 0.04 | 0.08 | 0.02 | 0.38 | 0.01 | | 0.72 |
| Rheuma | 0.009 | 0.17 | 0.01 | 0.07 | 0.01 | 0.36 | 0.01 | 0.30 | 0.01 | 0.68 | 0.009 | | 0.95 |
| Uveitis | 0.02 | 0.22 | 0.03 | 0.06 | 0.03 | 0.29 | 0.05 | 0.02 | 0.02 | 0.52 | 0.01 | | 0.83 |
| PSC | 0.007 | 0.59 | 0.006 | 0.52 | 0.01 | 0.58 | 0.007 | 0.89 | 0.01 | 0.81 | 0.02 | | 0.56 |
| Urinary stones (yes/no) | 0.03 | 0.009 | 0.02 | 0.03 | 0.03 | 0.01 | 0.02 | 0.05 | 0.02 | 0.58 | 0.02 | | 0.48 |
| Cholelithiasis (yes/no) | 0.03 | 0.001 | 0.009 | 0.26 | 0.03 | 0.008 | 0.01 | 0.26 | 0.04 | 0.01 | 0.02 | | 0.39 |
| Metabolic Bone Disease (yes/no) | 0.03 | 0.006 | 0.02 | 0.13 | 0.03 | 0.02 | 0.01 | 0.28 | 0.05 | 0.08 | 0.04 | | 0.31 |
| Absces (yes/no) | 0.03 | 0.001 | 0.03 | 0.001 | 0.03 | 0.006 | 0.02 | 0.08 | 0.04 | 0.04 | 0.07 | | 0.007 |
| Fistula (yes/no) | 0.02 | 0.001 | 0.01 | 0.03 | 0.03 | 0.02 | 0.01 | 0.43 | 0.05 | 0.02 | 0.09 | | 0.02 |
| F-calprotectin | 0.01 | 0.09 | 0.008 | 0.36 | 0.01 | 0.29 | 0.01 | 0.60 | 0.03 | 0.13 | 0.01 | | 0.62 |
| Albumin | 0.02 | 0.002 | 0.02 | 0.007 | 0.04 | 0.003 | 0.03 | 0.02 | 0.01 | 0.91 | 0.009 | | 0.91 |
| Leukocytes | 0.01 | 0.03 | 0.01 | 0.05 | 0.01 | 0.38 | 0.01 | 0.19 | 0.02 | 0.28 | 0.02 | | 0.64 |
| *Monocytes* | 0.01 | 0.03 | 0.008 | 0.40 | 0.02 | 0.13 | 0.01 | 0.48 | 0.02 | 0.69 | 0.01 | | 0.80 |
| *Neutrophils* | 0.01 | 0.07 | 0.01 | 0.07 | 0.02 | 0.21 | 0.01 | 0.59 | 0.02 | 0.51 | 0.02 | | 0.53 |
| PPI | 0.01 | 0.12 | 0.02 | 0.02 | 0.09 | 0.18 | 0.02 | 0.08 | 0.02 | 0.36 | 0.02 | | 0.45 |

## **Supplemental table 4**

| Supplemental Table 4: Significant differentially relative abundant ASVs between CD-EIM and CD-C | | | | | | | |
| --- | --- | --- | --- | --- | --- | --- | --- |
| ASV | **Mean**  **diff** | **Log2**  **FC** | **p.fdr** | **Phylum** | **Family** | **Genus** | **Species** |
| SV_36  SV_184  SV_239  SV_242  SV_34 | -832.2  -61.78  -47.57  19.42  212.66 | -9.7  -5.9  -5.6  4.2  7.7 | 0.017  0.003  0.028  0.013  0.011 | *Firmicutes*  *Firmicutes*  *Firmicutes*  *Firmicutes*  *Firmicutes* | *Lachnospiraceae*  *Lachnospiraceae*  *Lachnospiraceae*  *NA*  *Lachnospiraceae* | *Agathobacter*  *NA*  *NA*  *NA*  *Dorea* | *NA*  *NA*  *NA*  *NA*  *formicigenerans* |

## **Supplemental table 5**

| Supplemental Table 5: Significant differentially relative abundant ASVs between UC-EIM and UC-C | | | | | | | |
| --- | --- | --- | --- | --- | --- | --- | --- |
| ASV | M**e**a**n**  **diff** | **log2**  **FC** | **p.fdr** | **Phylum** | **Family** | **Genus** | **Species** |
| ASV_91 | -462 | -8.85 | 0.0039 | *Firmicutes* | *Ruminococcaceae* | *Faecalibacterium* | *NA* |
| ASV_48 | -425 | -8.73 | 0.0239 | *Bacteroidota* | *Bacteroidaceae* | *Bacteroides* | *vulgatus* |
| ASV_158 | -131 | -7.03 | 0.0007 | *Firmicutes* | *Lachnospiraceae* | *Agathobacter* | *NA* |
| ASV_192 | -110 | -6.78 | 0.0095 | *Firmicutes* | *Lachnospiraceae* | *Roseburia* | *NA* |
| ASV_204 | -97 | -6.60 | 0.0000 | *Bacteroidota* | *Bacteroidaceae* | *Bacteroides* | *fragilis/koreensis/kribbi/ovatus* |
| ASV_241 | -87 | -6.44 | 0.0316 | *Bacteroidota* | *Tannerellaceae* | *Parabacteroides* | *distasonis* |
| ASV_233 | -50 | -5.65 | 0.0000 | *Firmicutes* | *Lachnospiraceae* | *Lachnoclostridium* | *NA* |
| ASV_342 | -38 | -5.25 | 0.0004 | *Proteobacteria* | *Sutterellaceae* | *Sutterella* | *massiliensis/stercoricanis/wadsworthensis* |
| ASV_572 | -28 | -4.82 | 0.0245 | *Firmicutes* | *Lachnospiraceae* | *Lachnospira* | *NA* |
| ASV_337 | -26 | -4.70 | 0.0187 | *Firmicutes* | *Lachnospiraceae* | *NA* | *NA* |
| ASV_271 | -26 | -4.68 | 0.0207 | *Firmicutes* | *Lachnospiraceae* | *NA* | *NA* |
| ASV_274 | -17 | -4.11 | 0.0012 | *Actinobacteriota* | *Bifidobacteriaceae* | *Bifidobacterium* | *dentium/moukalabense* |
| ASV_168 | -6 | -2.62 | 0.0386 | *Firmicutes* | *Lachnospiraceae* | *NA* | *NA* |
| ASV_93 | 38 | 5.23 | 0.0155 | *Firmicutes* | *Lachnospiraceae* | *NA* | *NA* |
| ASV_235 | 52 | 5.70 | 0.0071 | *Firmicutes* | *Lachnospiraceae* | *NA* | *NA* |
| ASV_82 | 82 | 6.37 | 0.0305 | *Firmicutes* | *Lachnospiraceae* | *Blautia* | *NA* |
| ASV_80 | 262 | 8.04 | 0.0376 | *Firmicutes* | *Ruminococcaceae* | *Subdoligranulum* | *NA* |
